# Soft-Tissue Mobilization and Pain Neuroscience Education for Chronic Nonspecific Low Back Pain with Central Sensitization: A Prospective Randomized Single-Blind Controlled Trial

**DOI:** 10.3390/biomedicines11051249

**Published:** 2023-04-23

**Authors:** Jeongkeun Song, Hyunjoong Kim, Jihye Jung, Seungwon Lee

**Affiliations:** 1 Yes Home Rehabilitation Center, 370-32 Seoljuk-ro, Gwangju 61052, Republic of Korea; song950502@gmail.com; 2 Neuromusculoskeletal Science Laboratory, 306 Jangsin-ro, Gwangju 62287, Republic of Korea; hyun-joongkim@nmslab.org; 3 Institute of SMART Rehabilitation, Sahmyook University, 815 Hwarang-ro, Seoul 01795, Republic of Korea; jihye3752@gmail.com; 4 Department of Physical Therapy, College of Health and Welfare, Sahmyook University, 815 Hwarang-ro, Seoul 01795, Republic of Korea

**Keywords:** pain, neuroscience, patient education, central sensitization, low back pain

## Abstract

This study was conducted to demonstrate the therapeutic effect of soft-tissue mobilization (STM) combined with pain neuroscience education (PNE) for patients with chronic nonspecific low back pain with central sensitization. A total of 28 participants were recruited and randomly allocated to either the STM group (SMG) (*n* = 14) or the STM plus PNE group (BG; blended group) (*n* = 14). STM was applied twice a week for four weeks, with a total of eight sessions, and PNE was applied within four weeks, for a total of two sessions. The primary outcome was pain intensity, and the secondary outcomes were central sensitization, pressure pain, pain cognition, and disability. Measurements were made at baseline, after the test, and at 2-week and 4-week follow-ups. The BG showed significant improvement in pain intensity (*p* < 0.001), pressure pain (*p* < 0.001), disability (*p* < 0.001), and pain cognition (*p* < 0.001) compared to the SMG. This study demonstrated that STM plus PNE is more effective for all measured outcomes compared to STM alone. This finding suggests that the combination of PNE and manual therapy has a positive effect on pain, disability index, and psychological factors in the short term.

## 1. Introduction

Chronic nonspecific low back pain (CNLBP) accounts for 85–90% of chronic low back pain, and the underlying pathological mechanism remains unclear [1,2]. According to a study on the pathological mechanism [3], CNLBP can be classified as nonmechanical or mechanical pain. Notably, nonmechanical pain characteristics are suggested to be caused by central sensitization, which involves a wide range of changes in pain processing in the central nervous system.

A biopsychosocial approach is recommended to understand and manage central sensitization [4]. Based on this information, pain and pain-related behaviors should be considered the results of interactions between biological, psychological, social, and contextual factors. Pain neuroscience education (PNE) is an example of a biopsychosocial approach in the field of physical therapy [5,6]. PNE is a top-down educational therapy that changes patients’ beliefs based on a neurophysiological understanding of pain [7,8], and focuses on pain experience and cognition of chronic pain [9,10].

In a meta-analysis of the effects of PNE on kinesiophobia in patients with chronic pain, it was reported that a combined intervention of manual therapy and exercise therapy was effective in improving chronic pain and kinesiophobia [5,6,11]. Similarly, soft-tissue mobilization (STM) in CNLBP has been reported as a manual therapy that could help avoid short-term pain, disability, and fear [11]. STM is known to be safer and more effective than other manual therapy techniques or vigorous exercise [12,13].

Previous studies have confirmed that PNE and STM can improve pain, disability, and psychological factors related to CNSLBP or central sensitization [11,13,14]. This study aimed to investigate the effectAs of STM alone compared to STM plus PNE on pain, disability, and psychological factors in patients with CNLBP with central sensitization. Therefore, the hypotheses of the study are as follows: when the application of soft tissue mobilization to individuals with chronic back pain is combined with pain neuroscience education of the top-down approach to central sensitization, then pain, the disability index, and psychological factors will show different aspects depending on the period.

## 2. Materials and Methods

### 2.1. Study Design

This study was a prospective, parallel-arm, assessor-blinded, randomized, active-controlled trial. The trial protocol was conducted from August to October 2021 after pre-registration (No. KCT0006455).

### 2.2. Participants and Ethics

This study included patients who visited the Gwangju Heemang Hospital (Gwangju, Republic of Korea) in August 2021 with a primary complaint of low back pain (LBP). Patients were diagnosed with low back pain based on radiological examination, physician screening, and history taking. Among them, patients with CNLBP of an unknown cause were recruited through an in-hospital bulletin board announcement, excluding specific pathologies (infection, tumor, osteoporosis, fracture, structural deformity, inflammatory disorder, neuromuscular syndrome, and cauda equina syndrome) of known cause.

#### 2.2.1. Inclusion Criteria

The inclusion criteria were: male and female patients between the ages of 20 and 75 years who were diagnosed with CNLBP and had symptoms of >12 weeks; scored ≥28 points on the Korean version of the central sensitization inventory (CSI-K); and had an LBP of 3 or higher on the numeric pain rating scale (NPRS) within the last 7 days [1,15].

#### 2.2.2. Exclusion Criteria

Exclusion criteria included the following: a history of neck or back surgery within the past 3 years; a diagnosis of mental illness; patients with rheumatic disease, neurological disease, heart disease, respiratory disease, metabolic disease, endocrine disease, fibromyalgia, or chronic fatigue syndrome; patients with the same chronic pain syndrome; loss of skin sensation; skin inflammation or swelling on the back; and the use of painkillers or oral and systemic steroids for more than 10 days a month [16,17,18,19,20].

#### 2.2.3. Ethics

Before the trial started, participants were provided with information about the study and possible risk factors. Afterwards, the participants were asked to sign a consent form and were informed that they could withdraw their participation at any time. This study was approved by the Institutional Review Board of Sahmyook University (No. 2-1040781-A-N-012021090HR).

### 2.3. Sample Size

To calculate the sample size, the effect size f(V) for pain intensity was set at 0.25 based on a previous study [17] in which PNE and therapeutic exercise were applied to treat patients with CLBP in a parallel study. The G*power 3.1.9.6 (Franz Faul, Universitiat Kiel, Germany) was used to calculate sample size, and a total of 24 participants were set with a power of 0.80, a significance level of 0.05, two groups, four measurements, and a correlation of 0.5 between repeated measurements. The calculation formula to be assigned was established. Thus, 28 participants were recruited for the study, considering a possible dropout rate of 20%.

### 2.4. Randomization and Blinding

The study participants were randomly allocated to two groups, i.e., soft-tissue mobilization group (SMG) and blended group (BG; STM plus PNE) using a random allocation software (Isfahan University, Isfahan, Iran), with a four-digit number identification code. The investigators and participants were not blinded, and only the assessors were blinded to all measurements.

### 2.5. Interventions

All participants underwent STM two sessions per week for 40 min per session for four weeks. Additionally, the BG received two sessions (30–50 min) before and after receiving STM. All participants were controlled for factors influencing outcomes such as medical treatment, exercise, and supplements while the intervention was provided.

#### 2.5.1. Soft-Tissue Mobilization

Four STM techniques were used for STM, including transverse sliding of the lumbar muscles, thoracolumbar myofascial release, quadratus lumborum myofascial release, and psoas myofascial release [11,21].

Transverse sliding of the lumbar muscles was performed in prone position. The physical therapist performed transverse sliding along the paraspinal muscles using the elbow, three times on each side.

Thoracolumbar myofascial release was performed in the prone position. The physical therapist crossed and contacted the participant’s T12-L1 level and sacrum using their hands. After contact, care was taken not to slide over the skin or apply strong pressure to the tissue, which was carried out along the fascia for at least 5 min.

Quadratus lumborum fascia release was performed in the prone position. The physical therapist’s elbow was brought into contact with the participant‘s quadratus lumborum muscle above the iliac crest, and the participant‘s thigh was gripped and fixed with the opposite hand. Subsequently, the physical therapist’s elbow applied low-intensity pressure toward the participant’s spine and the hand located on the thigh was gently pulled downward. The technique took 7 min on each side.

The psoas fascia was released with the patient in the supine position. The physical therapist’s hand was brought into contact with the participant’s navel (3 cm on each side) and relaxation of the fascia was induced through horizontal sliding. This technique was performed 15 times on each side.

#### 2.5.2. Pain Neuroscience Education

The educational program was structured based on the pain neuroscience curriculum based on the revised Neurophysiology of Pain Questionnaire (rNPQ) included in “Pain Neuroscience Education” [17,22] (Table 1).

The first session was held in the hospital’s rehabilitation treatment room before the STM program started. The training was conducted through examples, metaphors, and verbal explanations using Power Point™ (Microsoft Corporation, Redmond, WA, USA) to enhance understanding [17]. After the education was completed, the participants were asked to fill out the rNPQ to check their degree of understanding of the educational content and to monitor knowledge change [23,24,25]. The rNPQ is a reliable questionnaire that can determine pain knowledge in a population with chronic pain [23]. The correct answers were fixed so that incorrect answers could be used as reference material in the second session. After education and preparation of the rNPQ were completed, a booklet containing the educational content was distributed to the participants and guided to read carefully at home [17,25].

The second session was held at the same place after the end of STM [17]. It was conducted by explaining incorrect items among participants’ rNPQ results again and answering additional questions after reading the booklet [23,25]. After the training was completed, the participants filled out the rNPQ again to confirm that they understood most of the information provided [23,25]. After the second session, the monitored group B had a correct answer rate of 96%, an incorrect answer rate of 2%, and a non-response rate of 2%. Similarly, the STM group had a correct answer rate of 97%, an incorrect answer rate of 1%, and a non-response rate of 2%.

### 2.6. Outcome Measures

The total study period was eight weeks, consisting of a four-week intervention period and a four-week follow-up period. Before the intervention was provided, participants had a baseline measurement, and after the intervention was completed, they received a post-test, follow-up at two weeks, and follow-up at four-week assessment as well.

#### 2.6.1. Primary Outcome Measure

The NPRS is a pain intensity rating scale consisting of an 11-point scale ranging from 0 (no pain) to 10 (worst pain). In this study, triple NPRS was applied as an evaluation method of NPRS, which calculates the overall average by receiving the patient’s highest and lowest pain scores for the current and previous 24 h [26]. The intraclass correlation coefficient (ICC) for the test-retest reliability of the triple NPRS was 0.61 to 0.77, and the minimal clinically important difference (MCID) was reported to be 2 points [27].

#### 2.6.2. Secondary Outcome Measures

The CSI is a comprehensive screening tool developed to evaluate central sensitization symptoms in patients with chronic pain and is an effective treatment outcome measurement tool [28]. CSI consists of two questionnaires, CSI A and CSI B, and the score is calculated only with CSI A. CSI A asks how often you experience each symptom for 25 items. Each item was answered on a scale from 0 (not at all) to 4 (always), with a total score ranging from 0 to 100. The test-retest reliability of the CSI was reported as *r* = 0.817, the internal agreement was reported as Cronbach’s α = 0.879 [29], and the minimal detectable change (MDC) was confirmed to be 5.092 in a study by Bid et al. [30] for CLBP patients. In this study, the CSI-K, which has been translated into Korean and has excellent validity and reliability, was used [31].

Pressure pain threshold (PPT) was measured using a digital algometer (Pain Test™ FPX 25 Algometer, Wagner Instruments, Greenwich, CT, USA) with a probe surface area of 1 cm^2^. Based on the study by Paulo et al. [32], who applied STM to patients with CLBP and evaluated PPT, the measurement site was set at a point 2 cm lateral to the midline between the spinous processes of L3 and L4 [20]. The evaluator marked the participant’s measuring point with a pen and gradually applied pressure of 9.8 N/cm^2^ (1 kgf/cm^2^) per second vertically to the skin on average until the participant complained of pain or discomfort [32]. If they complained of pain, the device was removed from the participant’s body and the digitized pressure was recorded in ‘Newton’. The average value was used for analysis by measuring three times at 30-second intervals. In a study by Jensen et al. [33], who studied the reliability of PPT for CLBP patients, the intra-rater reliability was reported as ICC = 0.83 for more experienced raters, and ICC = 0.72 for less experienced raters. This study showed ICC = 0.77 for more experienced evaluators and ICC = 0.84 for less experienced evaluators. The test-retest reliability was reported as ICC = 0.99 [34], and MCID was considered an increase of 15% or more [35].

The pain catastrophizing scale (PCS) is a common assessment tool used to measure pain catastrophizing [36] and evaluate exaggerated negative thoughts or feelings about pain [37]. The total number of items consists of 13 items, and the score for each item is calculated on a 5-point Likert scale, ranging from 0 (‘not at all’) to 4 (‘strongly agree’), ranging from 0 to 52 points in total. In this study, the Korean version of the PCS (K-PCS) was used, which reported excellent test-retest reliability (ICC = 0.79) and internal agreement (Cronbach’s α = 0.93) for all items [38]. It was confirmed that the MDC of the K-PCS was 10.28 points based on the total score for patients with chronic noncancer pain [38].

The Tampa scale of kinesiophobia-17 (TSK-17) is a self-report questionnaire frequently used to assess fear of movement [39]. There are a total of 17 items, and each item uses a 5-point Likert scale ranging from 1 = “disagree” to 4 = “strongly agree ”. The total score ranges from 17 to 68, with higher scores indicating greater fear of injury or recurrence. The test-retest reliability for TSK-17 is *r* = 0.29 to 0.69 [40], and the MCID for CLBP patients was reported to be 5.5 points [41].

The Korean version of the Roland Morris Disability Questionnaire (K-RMDQ) is designed for self-reporting disability due to LBP [42]. It consists of 24 items and reflects individual perceptions and limitations in various activities of daily living according to LBP [42]. Each item is worth one point, and you mark the item that is appropriate for your status. The total score ranges from 0 (no disability) to 24 (maximum disability), with a higher score indicating a higher level of pain-related disability. The reported MCID was three points [43].

### 2.7. Data Analysis

SPSS (Statistics 25 version, IBM Corp., Armonk, New York, USA) was used for data analysis. The mean and standard deviation of all collected data were calculated using descriptive statistics, and normal distribution was verified using the Shapiro–Wilk test. The general characteristics of the participants were verified for homogeneity using the chi-squared test and independent samples *t*-test. Mauchly’s test was performed for all variables measured according to the time point and the assumption of sphericity was satisfied.

Two-way repeated measures analysis of variance (RM ANOVA) was performed to examine the interaction between time and group. When the interaction was proven, an independent sample *t*-test was conducted as a post hoc test for differences between groups at each time point. The main effect of time was determined by performing one-way RM ANOVA. If a main effect of time was demonstrated, post hoc tests were conducted for differences at each time point relative to the baseline. The statistical significance level (α) for analysis of variance was set at 0.05. The significance level for the post hoc test was corrected using Bonferroni’s method, with the significance level (α) of the group comparison by time point being 0.013 and the significance level (α) of the post hoc test for the comparison between the time points being 0.017. In the ANOVA results, partial eta square ($\eta_{p}^{2}$) was used for effect size, and Cohen’s d was used for effect size according to the time point for each group.

## 3. Results

**Figure 1:**
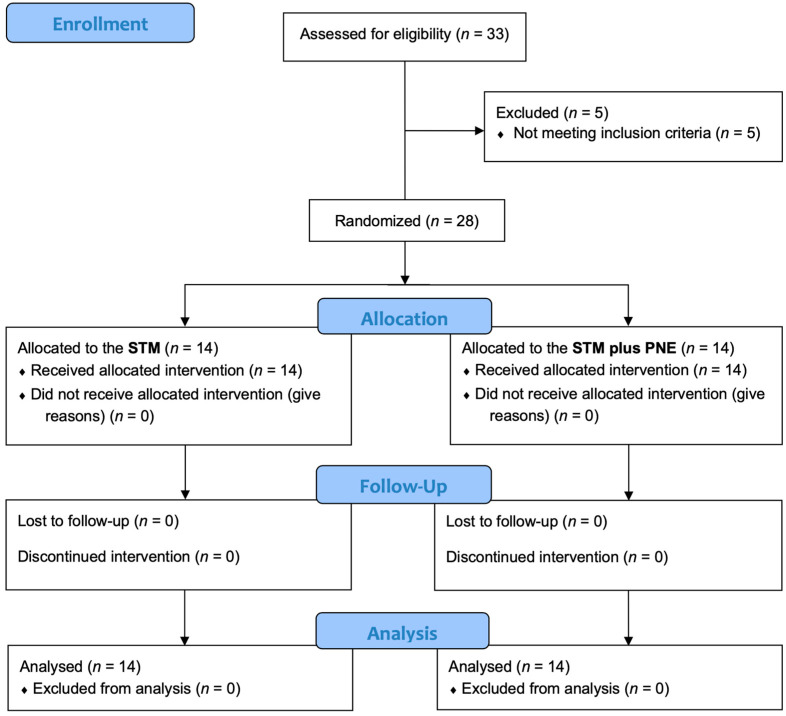
Study flow diagram. PNE: pain neuroscience education; STM: soft-tissue mobilization.

This study is a Consolidated Standards of Reporting Trials (CONSORT)-compliant randomized controlled trial. Figure 1 shows the CONSORT patient flow diagram. A total of 33 potential participants were recruited but 5 were excluded according to the eligibility criteria, resulting in a final enrollment of 28 participants.

### 3.1. Participants’ General Characteristics

Table 2 presents the general characteristics of the enrolled participants. No significant differences were found between the groups for any of the variables measured in the homogeneity test.

### 3.2. Pain Intensity

The NPRS showed an interaction between time and group (*F* = 14.951, *p* < 0.001, $\eta_{p}^{2}$ = 0.365). As a result of a post hoc test to determine the difference between the groups by time point, the BG showed significant improvement compared to SMG at 2 weeks and 4 weeks (*p* < 0.013) (Table 3).

**Figure 2:**
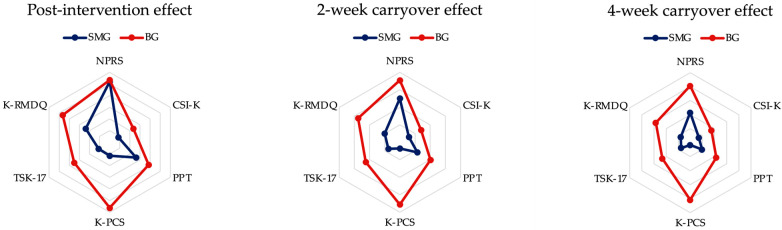
Radar chart of effect size comparison between groups by time point. BG, blended group; CSI-K, Korean version of the central sensitization inventory; K-PCS, Korean version of the pain catastrophizing scale; K-RMDQ, Korean version of the Roland Morris disability questionnaire; PPT, pressure pain threshold; TSK-17, Tampa scale of kinesiophobia-17; SMG, soft-tissue mobilization group.

The comparison of effect sizes between groups by measurement time point, post-intervention, and 2- and 4-week carryover effects all showed a larger effect size in the BG compared to the SMG (Figure 2) (Table 4).

### 3.3. Central Sensitization

The CSI-K showed an interaction between time and group (*F* = 33.829, *p* < 0.001, $\eta_{p}^{2}$ = 0.565); however, no time point showed a significant difference in Bonferroni’s post hoc test results (*p* > 0.013) (Table 3). Nevertheless, in the comparison of effect sizes between groups by measurement time point, post-intervention, 2-week carryover, and 4-week carryover effects all showed larger effect sizes in the BG than in the SMG (Figure 2) (Table 4).

### 3.4. Pressure Pain

The PPT showed an interaction between time and group (*F* = 46.518, *p* < 0.001, $\eta_{p}^{2}$ = 0.641). A post hoc test to determine the difference between the groups by time point showed a significant improvement in the BG compared to the SMG at the post-test, the 2-week, and the 4-week follow-up (*p* < 0.013) (Table 3). Similarly, a comparison of effect sizes between the two groups using the measured time points—post-intervention, 2-week carryover, and 4-week carryover effects—showed larger effect sizes in BG compared to the SMG (Figure 2) (Table 4).

### 3.5. Pain Cognition

The K-PCS showed an interaction between time and group (*F* = 62.178, *p* < 0.001, $\eta_{p}^{2}$ = 0.705). In the post hoc test results to determine the difference between groups by time point, BG showed significant improvement compared to SMG at the post-test, 2-week, and 4-week follow-up (*p* < 0.013) (Table 3). Similarly, comparisons of effect sizes between groups by measurement time points—post-intervention, 2-week carryover, and 4-week carryover effects—all showed larger effect sizes in BG compared to the SMG (Figure 2) (Table 4).

Additionally, TSK-17 showed an interaction between time and group (*F* = 23.098, *p* < 0.001, $\eta_{p}^{2}$ = 0.470). In the post hoc test to determine the difference between groups by time point, BG showed significant improvement compared to SMG at the post-test, 2-week, and 4-week follow-up (*p* < 0.013) (Table 3). Similarly, comparisons of effect sizes between groups by measurement time points—post-intervention, 2-week carryover, and 4-week carryover effects—all showed larger effect sizes in BG compared to SMG (Figure 2) (Table 4).

### 3.6. Disability

The K-RMDQ showed an interaction between time and group (*F* = 26.466, *p* < 0.001, $\eta_{p}^{2}$ = 0.504). As a result of a post hoc test to determine the difference between groups by time point, BG showed significant improvement compared to SMG at 2 weeks and 4 weeks (*p* < 0.013) (Table 3). 

In the comparison of effect sizes between groups by measurement time points—post-intervention, 2-week carryover, and 4-week carryover effects—all showed a larger effect size in the BG than in the SMG (Figure 2) (Table 4).

## 4. Discussion

This study is the first to combine STM and PNE for treating patients with CNLBP with central sensitization. By comparing the effects of STM alone and STM plus PNE, this study aimed to determine their effects on pain intensity, central sensitization, pressure pain, pain cognition, and disability. The results showed a significant interaction between time and group for all variables (*p* < 0.001). In the post hoc tests, a significant difference was found in all variables except CSI-K at follow-up (2-week and 4-week) (*p* < 0.013). BG had a larger effect size than SMG in the comparison of effect sizes between the groups.

In the NPRS, a primary outcome measure, only the BG showed a decrease of two or more points in the MCID. These results suggest that the addition of PNE is more effective in helping control pain intensity. This may be due to the fewer fear-avoidance beliefs observed in patients with high pain knowledge. Notably, fear-avoidance beliefs are positively correlated with pain intensity [44,45]. In addition, in this study, rNPQ was monitored for the accurate delivery of pain knowledge and was constantly provided. A large effect size was observed in the post-intervention effect size but no statistically significant results were found. Similar to a previous study [46] on additional PNE, the immediate change in pain reconceptualization might be delayed rather than an immediate effect. 

In the secondary outcome measures, CSI-K showed a significant interaction between time and group but no significant time point was found in the post hoc test. However, when compared to the reported MDC, there was a significant difference between the two groups. Considering the difficulty in suggesting a clear mechanism, and the fact that Nijs et al. [47] focused on long-term rather than short-term changes for the management of central sensitization (as shown in Figure 2 and Table 4), the increased effect size seems to be partially consistent with these results. In addition, all patients showed significant improvements in PPT over time. Both groups met the reported MCID score (≥15%). Interestingly, there is a positive correlation between the functional connectivity of the frontal and parietal lobes. This is relevant as the frontal–parietal lobe network plays an important role in anticipation-induced pain control [48,49]. Therefore, based on evidence that changes in pain perception due to pain education form relatively high expectations for recovery [50], the elevated expectations caused by PNE caused an increase in PPT by increasing functional connectivity in the frontal-parietal lobe network. 

In pain cognition, both the K-PCS and TSK-17 showed an interaction between time and group. In the post hoc test of the post-test and follow-up after 2 and 4 weeks, the BG showed a positive improvement compared to the SMG. As if indicating the effect of additional PNE, there was a meaningful change only in the BG in the reported MCID. It has been reported that reconceptualization through an increase in knowledge about pain through PNE shows a positive improvement in catastrophizing pain, which could lead to improvement in kinesiophobia by gradually lowering fear and inducing movement [48,49,50]. Similarly, the significant improvement in the K-RMDQ results of this study is because the disability index correlates with fear avoidance [51].

These results demonstrate the positive effect of PNE plus STM, compared to the effect of PNE alone. PNE has been reported to last for 3 months due to long-term retention of pain knowledge, even if only given once [6]. These results are consistent with the increasing effect size over time shown in Figure 2 and Table 4. Summarizing through the pain-tension-fear cycle [52,53], the decrease in pain intensity led to a decrease in PPT due to lower tension, and the positive effect of additional PNE was shown by reducing fear according to pain catastrophe and improvement of kinesiophobia.

In addition, according to the effect size results of this present study, the effect of STM showed more than a moderate effect in all variables except for PCS-K. As a basis for interpreting the results of this study, it has been reported that STM, which is included in massage therapy, induces a decrease in cortisol and an increase in serotonin, dopamine, and oxytocin in response to stressful experiences [51,52,53]. Similarly, an increase in the amount of change in superficial sensation due to the neuroplastic effect leads to an increase in the amount of change in PPT in the same spinal segment [54], and the decrease in superficial sensation could continue even after treatment is completed [55].

These findings may provide options for physical therapists to consider cognitive factors of pain as well as biological interventions when managing patients with central sensitization. In further studies, physical therapists should consider applying 1:1 sessions and longer follow-ups to provide maximum benefit to individual patients [56]. Likewise, studies in the direction of enhancing understanding of education and cognition of being aware of pain are needed. Furthermore, it is necessary to consider acquiring pedagogical knowledge about patient education in higher education institutions such as universities. The limitations of this study are as follows: first, only short-term follow-up was conducted; second, demographic factors such as education and age of the participants were not considered; third, the single-centered process might limit generalization; fourth, there are still conflicting opinions on how to deliver PNE. However, the strengths of this study showed clinically significant changes in most variables. Considering the design and limitations of this study, more positive improvements could be expected.

## 5. Conclusions

In conclusion, STM plus PNE may be provided as a sufficient treatment option for positive improvement in pain, pain cognition, and disability in patients with CNLBP with central sensitization when compared to STM alone.

## Figures and Tables

**Table 1:** Pain neuroscience education.

Chapter	Topic	rNPQ
1	Traditional and Old Pain Models	
2	Changing Beliefs about Pain	
3	Input Mechanism: Tissues	2, 3, 4, 7, 8, 10, 12
4	Input Mechanism: Environment	11
5	Input Mechanism: Peripheral Neurogenic	6
6	Processing Mechanism: Spinal Cord, Dorsal Horn, and Second-order Neurons	5, 9
7	Processing Mechanism: Brain, The Pain Neuromatrix, and Functional Changes in the Brain	1, 3, 9, 10, 12
8	Processing Mechanism: The Pain Neuromatrix, Yellow Flags, and“Personalization” of the Pain Experience	9, 11, 12
9	Output Mechanisms: The Stress Response, Endocrine System, and Immune System	6
10	Plasticity and Merging of Systems	3, 7, 10, 12

rNPQ, revised Neurophysiology of Pain Questionnaire.

**Table 2:** Participants’ General Characteristics.

Variables	SMG (*n* = 14)	BG (*n* = 14)	Χ^2^/t	*p*
	Mean ± SD	Mean ± SD		
Sex (male, n)	8	9	0.150	0.699
Age (year)	45.21 ± 12.32	45.64 ± 12.33	−0.092	0.865
Height (cm)	167.55 ± 7.27	168.50 ± 6.05	−0.376	0.483
Weight (kg)	68.09 ± 14.85	64.99 ± 8.71	0.675	0.159
BMI (kg/m^2^)	24.06 ± 3.72	22.87 ± 2.68	0.970	0.236
Duration of disease (weeks)	41.07 ± 12.47	34.43 ± 13.64	1.345	0.190

BG, blended group; SD, standard deviation; SMG, soft-tissue mobilization group.

**Table 3:** Differences between groups by measurement time point for pain intensity, central sensitization, pressure pain, pain cognition, and disability.

Variables		Baselines	Post-Test	Follow-Up at 2 Weeks	Follow-Up at 4 Weeks	Time	Time × Group ^(a)^	
						F	F	Effect Size ^(b)^
Pain intensity								
NPRS ^(c)^	SMG	4.66 ± 0.92	2.59 ± 0.64 *	2.64 ± 0.67 *	2.93 ± 0.67 *	277.411 ^‡^	14.951 ^‡^	0.365
	BG	4.73 ± 0.90	2.40 ± 0.85 *	1.95 ± 0.65 *^†^	1.78 ± 0.50 *^†^			
Central sensitization								
CSI-K ^(c)^	SMG	39.93 ± 8.07	36.57 ± 7.45 *	35.43 ± 6.98 *	34.79 ± 6.49 *	241.550 ^‡^	33.829 ^‡^	0.565
	BG	41.00 ± 7.39	33.07 ± 6.11 *	31.64 ± 5.84 *	29.57 ± 5.69 *			
Pressure pain								
PPT	SMG	29.23 ± 8.74	40.36 ± 8.32 *	39.18 ± 8.56 *	37.47 ± 8.39 *	315.334 ^‡^	46.518 ^‡^	0.641
	BG	33.13 ± 11.95	52.90 ± 8.21 *^†^	54.13 ± 8.58 *^†^	54.89 ± 7.98 *^†^			
Pain cognition								
K-PCS ^(c)^	SMG	30.93 ± 5.01	27.93 ± 5.27 *	28.86 ± 6.49	30.00 ± 6.30	109.980 ^‡^	62.178 ^‡^	0.705
	BG	29.86 ± 3.80	20.14 ± 3.08 *^†^	18.57 ± 2.38 *^†^	17.57 ± 1.91 *^†^			
TSK-17 ^(c)^	SMG	40.86 ± 4.93	38.43 ± 3.78 *	37.43 ± 3.99 *	37.57 ± 3.63 *	98.456 ^‡^	23.098 ^‡^	0.470
	BG	39.21 ± 5.12	31.57 ± 3.41 *^†^	29.93 ± 2.84 *^†^	29.50 ± 3.11 *^†^			
Disability								
K-RMDQ ^(c)^	SMG	9.07 ± 2.76	6.29 ± 1.86 *	6.64 ± 2.02 *	7.14 ± 2.07 *	154.778 ^‡^	26.466 ^‡^	0.504
	BG	9.71 ± 2.46	5.07 ± 1.38 *	4.36 ± 1.22 *^†^	4.21 ± 1.19 *^†^			

Values are presented as mean ± standard deviation unless otherwise indicated. ^(a)^ Two-way repeated-measures analysis of variance, ^(b)^ partial eta squared, ^(c)^ lower scores are better. BG, blended group; CSI-K, Korean version of the central sensitization inventory; K-PCS, Korean version of the Pain Catastrophizing Scale; K-RMDQ, Korean version of the Roland Morris disability questionnaire; PPT, pressure pain threshold; TSK-17, Tampa scale of kinesiophobia-17; SMG, soft-tissue mobilization group. * *p* < 0.017, statistically significant difference compared with baseline. ^†^ *p* < 0.013, each group difference at each point measurement with Bonferroni correction. ^‡^ *p* < 0.001, statistically significant difference.

**Table 4:** Comparison of effect size between groups by time point.

	NPRS	CSI-K	PPT	K-PCS	TSK-17	K-RMDQ
Post-intervention effect; post-test minus baseline						
SMG	2.602	0.432	1.305	0.583	0.553	1.185
BG	2.669	1.170	1.928	2.807	1.757	2.324
2-week carryover effect; follow-up at 2 weeks minus baseline						
SMG	2.504	0.596	1.150	0.357	0.764	1.004
BG	3.547	1.405	2.018	3.561	2.244	2.758
4-week carryover effect; follow-up at 4 weeks minus baseline						
SMG	2.152	0.702	0.962	0.163	0.759	0.791
BG	4.062	1.732	2.142	4.085	2.295	2.844

BG, blended group; CSI-K, Korean version of the central sensitization inventory; K-PCS, Korean version of the Pain Catastrophizing Scale; K-RMDQ, Korean version of the Roland Morris disability questionnaire; PPT, pressure pain threshold; TSK-17, Tampa scale of kinesiophobia-17; SMG, soft-tissue mobilization group.

## Data Availability

Not applicable.
